# Small RNA Pathways in Plants

**DOI:** 10.1371/journal.pbio.0020107

**Published:** 2004-02-24

## Abstract

xx

Since small RNA molecules were discovered just over ten years ago, it's become clear that these once overlooked bits of genetic material play a decidedly large role in controlling gene expression. Though typically just 21 to 24 nucleotides long, small RNAs regulate a diverse array of cellular processes, from developmental patterning and genome rearrangement to antiviral defense. They typically accomplish these tasks by targeting specific nucleotide sequences to shut down gene expression.[Fig pbio-0020107-g001]

**Figure pbio-0020107-g001:**
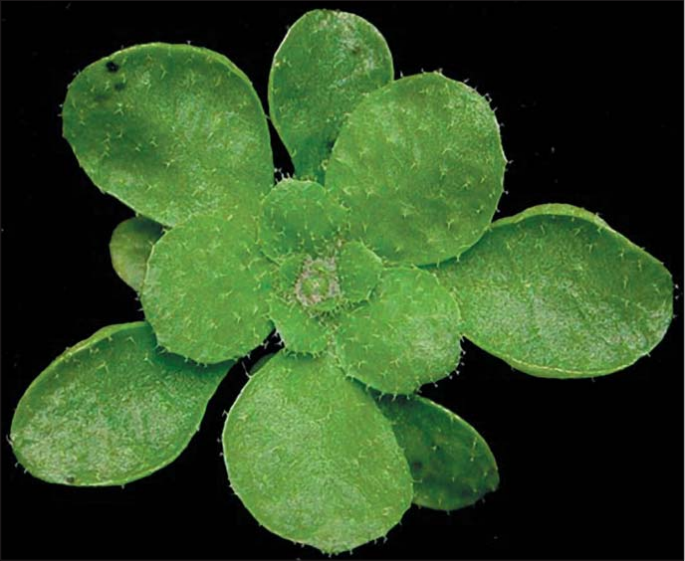
A. thaliana at the rosette stage

Found in both plants and animals, small RNAs come mainly in two classes—microRNA (miRNA) and short interfering RNA (siRNA). miRNAs arise from nonprotein-coding transcripts that adopt extended “fold-back” structures, which are then cleaved by enzymes called Dicer or Dicer-like (DCL). siRNAs, on the other hand, arise from perfectly base-paired double-stranded RNA, which are also cleaved by Dicer. Some siRNAs require enzymes called RNA-dependent RNA polymerases (RdRp). miRNAs and many types of siRNAs function post-transcriptionally—that is, they affect genes that have been expressed, or transcribed, into RNA—to guide cleavage or prevent translation into protein. In plants and some animals, this post-transcriptional RNA interference (RNAi) acts as an adaptive antiviral response, among other things. siRNAs can also “silence” gene expression by altering chromatin—the DNA-protein complex into which chromosomes assemble—and preventing transcription. It is thought that chromatin silencing acts as a genome defense mechanism, guarding against potential damage from mobile genetic elements or invasive DNA (say, from a virus) by keeping genes in the tightly coiled, and thus inaccessible, “heterochromatic” state.

While much remains to be learned about the mechanisms and pathways that govern small RNAs, it's becoming clear that they add an important layer of regulation and flexibility to gene expression. Now a team led by James Carrington at Oregon State University and Steve Jacobsen at the University of California at Los Angeles demonstrates that plants have evolved multiple systems to produce distinct classes of small RNAs with specialized regulatory and defensive functions. The first generates miRNAs; the second produces siRNAs that regulate chromatin structure; and the third generates siRNAs in response to viral infections. Each system requires a unique spectrum of functions of three different DCL proteins; the siRNA systems each function in coordination with one of several RdRp proteins. The researchers propose that the expansion and subsequent diversification of these proteins, which occurred in plants but not in many animals, has contributed to the diversification of specialized small RNA-directed pathways.

Working in Arabidopsis thaliana, a favorite model organism for plant biologists, Zhixin Xie et al. analyzed a series of mutants with nonfunctional *dcl* and *rdr* genes, as well as a few other mutants of interest, to determine how the small RNAs responded to loss of these proteins. Two mutations (one in a *dcl* gene and one in another gene) affected the miRNAs, either impairing their function or reducing their populations. None of the RdRp proteins had any effect on miRNAs. The researchers performed the same type of genetic analyses on siRNAs and found that a different DCL mutant caused a reduction in one class of siRNAs and that an RdRp mutant nearly eliminated these populations of siRNAs.

The diversity of siRNAs produced by the Arabidopsis genome reveals an important role in genome maintenance, expression, and defense, the authors conclude. Given that large numbers of siRNAs arise from highly repeated sequences—such as those introduced by viruses or mobile genetic elements—it may be that the cell senses such “invasive” sequence duplication events and enlists siRNAs to run interference by silencing these potentially damaging sequences. In this way, chromatin-associated siRNAs may offer an additional line of defense against invasive sequences, on top of that offered by post-transcriptional RNAi—a dual adaptive advantage since a fast-spreading virus or over-proliferating transposon (also known as a jumping gene) could wreak havoc on a plant population.

Whatever other roles small RNAs may play in genome regulation—they have also been implicated in regulating growth and development—their primary responsibility appears to be blocking gene expression. Whether they accomplish that by controlling chromosome activity to prevent gene transcription or by inhibiting or degrading RNA transcripts to block translation into protein, small RNAs appear to make wide-ranging contributions to the overall gene expression program of the cell.

